# SNP Identification from RNA Sequencing and Linkage Map Construction of Rubber Tree for Anchoring the Draft Genome

**DOI:** 10.1371/journal.pone.0121961

**Published:** 2015-04-01

**Authors:** Jeremy R. Shearman, Duangjai Sangsrakru, Nukoon Jomchai, Panthita Ruang-areerate, Chutima Sonthirod, Chaiwat Naktang, Kanikar Theerawattanasuk, Somvong Tragoonrung, Sithichoke Tangphatsornruang

**Affiliations:** 1 National Center for Genetic Engineering and Biotechnology, 113 Thailand Science Park, Paholyothin Road, Khlong Nueng, Khlong Luang, Pathumthani, 12120, Thailand; 2 Rubber Research Institute of Thailand (RRIT), Department of Agriculture, Ministry of Agriculture and Cooperatives, 50 Phaholyothin Road, Chatuchack, Bangkok, 10900, Thailand; Temasek Life Sciences Laboratory, SINGAPORE

## Abstract

*Hevea brasiliensis*, or rubber tree, is an important crop species that accounts for the majority of natural latex production. The rubber tree nuclear genome consists of 18 chromosomes and is roughly 2.15 Gb. The current rubber tree reference genome assembly consists of 1,150,326 scaffolds ranging from 200 to 531,465 bp and totalling 1.1 Gb. Only 143 scaffolds, totalling 7.6 Mb, have been placed into linkage groups. We have performed RNA-seq on 6 varieties of rubber tree to identify SNPs and InDels and used this information to perform target sequence enrichment and high throughput sequencing to genotype a set of SNPs in 149 rubber tree offspring from a cross between RRIM 600 and RRII 105 rubber tree varieties. We used this information to generate a linkage map allowing for the anchoring of 24,424 contigs from 3,009 scaffolds, totalling 115 Mb or 10.4% of the published sequence, into 18 linkage groups. Each linkage group contains between 319 and 1367 SNPs, or 60 to 194 non-redundant marker positions, and ranges from 156 to 336 cM in length. This linkage map includes 20,143 of the 69,300 predicted genes from rubber tree and will be useful for mapping studies and improving the reference genome assembly.

## Introduction

Assembly of a high quality reference genome, despite the rapid advances and falling costs in high throughput sequencing, remains a time consuming and costly procedure. A common and effective approach is to produce a library of insert clones (BAC, Fosmid, etc.), sequence them and de novo assemble each one, often these do not result in a single sequence and require additional work to order and close gaps [1]. Overlapping inserts are then merged resulting in generally large scaffolds which can be placed using flourescence in situ hybridisation, optical mapping or linkage mapping. This approach was taken for the genomes of rice [2], maize [3], barley [4] and wheat [5] and have resulted in the public availability of high quality genomes. A cheaper and less time consuming approach is to shotgun sequence the entire genome and de novo assemble the whole genome at once. This approach tends to result in heavily fragmented sequence, but generates usable data that can be placed into physical order using existing linkage maps or a closely related species. For example the Sorghum genome used a whole genome shotgun approach coupled with the use of insert libraries and linkage maps [6].

*Hevea brasiliensis*, or rubber tree, is an important crop species that accounts for the majority of natural latex production. Sequence and annotation of its nuclear genome [7], plastid genome [8] and mitochondrial genome [9] are important tools for genetic improvement and understanding of desirable traits. Rubber tree is an outbreeding species that is mainly insect pollinated [10]. Natural latex is harvested from rubber tree by scoring a downward spiral cut into the lactiferous vessels of the bark allowing natural latex to flow for collection. Trees are generally harvested every three days starting from when the tree is 5–7 years old with peak latex production occurring when the trees are 12–15 years old. This means selective breeding strategies are slow to achieve notable improvement and a molecular based approach could result in faster yield improvements. Research to date has focused on QTL mapping [11–14] and gene discovery [15–19], however, without a quality reference genome it is difficult to locate the underlying genes from the QTL studies which limits the potential of marker assisted selection for crop improvement. The availability of a polished reference genome for the rubber tree would greatly improve the ability to identify causative genes from within QTL regions and lead to faster crop improvement strategies.

The rubber tree nuclear genome consists of 18 chromosomes, is roughly 2.15 Gb, and filled with repeat sequences that make up around 78% of the genome [7]. This makes the rubber tree genome one of the more difficult genomes to sequence. A draft sequence of the rubber tree genome became available in 2013 using a whole genome shotgun sequencing approach. Despite the genome assembly using data from three different sequencing platforms, 454, Illumina and SOLiD and multiple library types, single end plus 200 bp, 500 bp, 8kb and 20 kb paired end reads, the large number of repeat elements resulted in a heavily fragmented genome. Prior to the scaffolding step the library consisted of 1,223,366 contigs ranging from 200 to 46,694 bp, scaffolding reduced the total assembly to 1,150,326 scaffolds and contigs ranging from 200 to 531,465 bp. Linkage map information [20] was incorporated into the rubber tree genome allowing for 143 scaffolds to be anchored [7]. The anchored contigs ranged from 590 to 431,849 bp, however, the largest scaffold and many other large scaffolds remain unplaced, thus there is still much room for improvement.

We have performed transcriptome sequencing (for review see [21]) of 6 rubber tree varieties with the aim of identifying SNPs. RNA-seq is the high throughput sequencing of all RNA that exists within the sample. RNA-seq is useful for generating transcriptome data for new species and for comparing expression levels between individuals. The advantage using RNA-seq for SNP identification is that many of the discovered SNPs are within coding genes and therefore have a greater potential to result in phenotypic variance than SNPs identified from genomic sequence [22]. We then used these SNPs for target capture and sequencing in a mapping population of 151 rubber trees, generated a linkage map and incorporated it with the existing linkage map information to further the rubber tree genome effort.

## Methods

### Plant materials and RNA sequencing

Samples from young leaf and shoot apical meristem from six varieties of rubber tree (varieties BPM 24, RRII 105, RRIC 110, PB 235, RRIT 251 and RRIM 600) were collected when the trees were roughly 18 years of age, DNA and RNA were extracted. RNA was sequenced for each sample on an Illumina HiSeq2000 using a 101 bp paired end method, with the exception of RRIM 600 which used a 75 bp single end method, as previously described in Shearman *et al*. [9].

The RNA-Seq reads (DDBJ: SRR1649353, SRR1649350, SRR1649349, SRR1649347, SRR1649351, SRR1649346 for BPM 24, RRII 105, RRIC 110, PB 235, RRIT 251 and RRIM 600, respectively) were aligned to the contigs of the rubber tree draft reference genome (GenBank: GCA_000340545.1) using TopHat v2.0.9 [23] calling Bowtie2 v2.1.0 [24] using default settings. The Genome Analysis Toolkit [25] Haplotype Caller (GATK, version 3.1-1-g07a4bf8), following the GATK best practices workflow for SNP and InDel calling on RNA-seq data (with the exception that we used TopHat instead of STAR as the aligner), was used to call SNPs in the 6 rubber tree varieties resulting in a multi-sample variant call format (VCF) file. Variant calls with a quality less than 40 and a combined-sample read depth less than 60 were excluded.

We followed the EVM pipeline method described in Rahman *et al*. [7] to create a genome annotation file for the published reference contig sequences. In addition, the pipeline program Maker was used to generate another genome annotation file [26]. The number of RNA-seq reads that were captured by each of the annotation files were determined using the python program HT-Seq [27]. From these two annotation files we produced a set of genes predicted by both methods (intersection) by identifying genes that were annotated to the same location in both files and sub-setting the Maker gene set (as these appeared to include start and stop codons more often). We also produced a non-redundant set of genes that were predicted by at least one of the methods (union) by adding any genes identified only by EVM to the set of genes identified by Maker. Both gene sets were then annotated using three databases: PlantGDB, SWISS-PROT and STRING, to obtain gene name and function information using an evalue cut-off of 1e-06. The degree to which the gene set matched the total RNA-seq mapping pattern was determined by counting how many of the RNA-seq reads mapped within the predicted gene locations. The number of genes with a functional annotation were counter per gene set, genes that were annotated as a transposon/retroelement, predicted protein or putative uncharacterised protein were not considered as a functional annotation. We then used these files to determine the effect of variants from the RNA-seq data using the program SNPEff v3.4e [28].

### DNA extraction, probe design and targeted enrichment sequencing

Samples were collected from a population of 149 F1 rubber tree offspring from a cross between RRIM 600 and RRII 105, DNA was extracted as previously described in Shearman *et al*. [9]. The genetic background of RRIM 600 and RRII 105 are described in [29]. At the time of capture probe design the rubber tree genome had not yet been released and we had some genomic sequence data of rubber variety BPM 24 (described in [9]), so the RNA-seq data was aligned to this using the same work flow as above with the exception that the reference was our own draft assembly of BPM 24. The resulting list of SNPs were filtered (as above) to select SNPs that were informative for the rubber tree varieties RRII 105 and RRIM 600 to be used for target enrichment capture probes. Capture probes were designed and synthesized by Life Technologies for the Ion TargetSeq Custom Enrichment Kit (Life Technologies, Grand Island, NY, USA) to target 10,317 SNPs from 8,395 contigs covering a total of 3.6 Mb of genomic DNA (S1 Dataset). Target capture and sequencing was performed in the parental varieties and the 149 offspring. For targeted enrichment sequencing, 500 ng of each DNA sample were fragmented enzymatically using the Ion Shear Plus Reagents (Life Technologies, Grand Island, NY, USA) and ligated to the barcoded adapters. Hybridization with the capture probes was performed according to the Ion TargetSeq Custom Enrichment Kit protocol. The adapter-ligated, enriched fragments were used to construct libraries, which were then sequenced on the Ion Torrent Proton System (Life Technologies, Grand Island, NY, USA) using the PI chip. The target probes were blasted against the published rubber tree reference genome once it became available.

### Target seq SNP calling

Raw reads were processed with the standard Ion Proton software (Life Technologies, Grand Island, NY, USA) to de-multiplex and trim adapter and barcode sequences. Clean reads were mapped to the rubber tree reference genome using the Ion software TMAP and the variants were called using GATK. The read uniformity distribution was calculated using the coverageAnalysis plugin from the Ion torrent suite software package (Life Technologies, Grand Island, NY, USA). The SNP calls from GATK were filtered to replace with a no-call any calls that had a read depth less than 20 or failed a binomial test for allele read depth, SNPs with less than 80% of samples called after call replacement were dropped. SNPs with a no-call in one or both of the parents were excluded, in addition any SNP where offspring had an allele not present in either parent were dropped. Finally, SNPs with abnormal segregation (χ2 test p-value < 1e-05) were also excluded.

### Construction of the linkage map

SNPs that passed the above selection criteria were used to construct linkage maps using Lep-MAP [30] with LOD score thresholds of 8–18. The maps were compared to the microsatellite based linkage map in Rahman *et al*. [7] to identify common contigs and their placement on each map. The map that most resembled the microsatellite based map and the chromosome structure (n = 18) was chosen. Linkage group numbering of the chosen map was updated using this overlap so that our linkage groups correspond directly to those in the literature. We identified which contig contained the microsatellite marker that was used to anchor the scaffolds [7] by blasting the miscrosatellite primer sequences against the reference genome. Blast match results were then filtered to select primers where the match included one or both of the 3`terminal bases and at least 80% of the entire primer sequence treating the forward and reverse primers separately. Primer pairs were then filtered to select only pairs that blasted in a forward-reverse (or vice-versa) orientation and produced a unique product of 500 bp or less for cases where the blast match was on the same contig. Cases where the blast match for the forward and reverse primers were on separate contigs were filtered to select primers with a 100% match for at least 70% of the length of the primer and for the blast match of each primer to fall within 300 bp of the start or end of the contig, primer pairs with multiple hits were excluded. The final linkage map was plotted using the R package ggbio [31].

## Results and Discussion

### Genome annotation

The genome annotation file created following the method of Rahman *et al*. [7] produced 62,981 predicted genes from 53,902 contigs and the annotation file created by the maker pipeline produced 26,144 genes from 22,640 contigs. The common set of genes predicted by both pipelines (intersect) contained 20,639 genes from 17,896 contigs (S2 Dataset) and the set of genes predicted by either (union) contained 69,300 genes from 58,172 contigs (S3 Dataset and S4 Dataset). The quality of the annotation files was assessed based on the number of genes that received an annotation through comparison to gene databases (S1 Table and S2 Table) and how closely the gene set matched the RNA-seq mapping pattern. The Union set of predicted genes had 85% of the genes match a database sequence, 59.4% of the predicted genes with a functional annotation and included 72.4% of the RNA-seq reads. The intersect set had 95.4% of the genes match a database sequence, 55.4% of the genes with a functional annotation and included 46% of the RNA-seq reads. The similar rate of gene annotation between the two sets suggests that neither is better than the other, but as the union set of genes includes the highest number of genes and RNA-seq reads it more closely represents the transcriptome so this set of genes was used for all additional analysis. Approximately 1.1% and 1.6% of the sequences in the intersect and union gene sets, respectively, were annotated as retroelement or transposon, but were not excluded as it is unknown whether or not they are functional.

### RNA-Seq and SNP calling

We sequenced the transcriptomes from 6 rubber tree varieties to develop a large number of coding SNP markers across the genome. Samples BPM 24, RRII 105, RRIT 110, PB 235 and RRIT 251 each had between 62 and 82 million paired end reads while sample RRIM 600, which was sequenced at an earlier time, had 17 million single end reads (S3 Table). The lower read number for RRIM 600 meant that this sample could be called for a fewer number or loci but did not affect the overall number of loci called. The RNA-seq reads mapped to 160,782 contigs from the published reference genome, however, 23,333 of these contigs accounted for 95% of the total number of reads. These 23,333, contigs overlap with 20,497 contigs from the union gene set annotation file. The 23,333 contigs include 123.5 Mb of sequence and 78.7% of the large (> 10 kb) contigs, yet only 607 have been placed into linkage groups which indicates that linkage mapping with coding SNPs could be of significant benefit in placing the rubber tree coding sequences into linkage groups. Relative to the reference sequence, we found 368,594 variants (S5 Dataset), consisting of 340,631 SNPs, which included 1,235 tri-allelic and 3 quad-allelic SNPs, 8,293 deletions, 17,272 insertions and 2,398 complex variants (where alternate alleles included a combination of insertion, deletion and SNP variants).

The frequency of single nucleotide substitutions found in the rubber tree transcriptome was approximately 1 in 270 nucleotides (using the combined length of the union gene set). The density of SNPs detected in the rubber tree is consistent with what has been reported in other plant species, such as maize (1 SNP/204 bp [32]), soybean (1 SNP/490 bp [33]) and pea (1 SNP/540 bp [34]). Of the 340,631 variations discovered, 195,913 (58%) were transitions (C/T or A/G) and 143,480 (42%) were transversions (A/C, A/T, C/G or G/T). In total, 154,500 of the 368,594 variants were within the intersect gene set and 251,952 were within the union gene set. The remaining 116,642 variants are from RNA sequence that did not get identified as a gene by either of the gene annotation pipelines. When the locations of the predicted genes from the union gene set were extended by 1 kb each side 335,334 (91%) of the variants fell within the extended gene locations, this suggests that those variants are in 3`and 5`untranslated regions. The remaining 33,260 variants are most likely explained by the high number of genes (64%) that are annotated with a start site less than 1kb along their respective contigs. This is further supported by the low number (20,241) of variants identified as being in the 3`or 5`untranslated region by SNPeff. The percentage of non-synonymous SNPs was 41.4%, and the majority of those were conservative mutations (S4 Table). The percentage of non-synonymous substitutions in rubber tree was lower than those reported in tomato (46%) [35] and Arabidopsis (45%) [36]. However, the fragmented nature of the reference genome means that the SNPeff results should be considered a prediction.

### Target enrichment and SNP calling

Sequence capture and sequencing of the parents and 149 offspring resulted in 1.5 to 21 million reads per sample with a median read count of 5.7 million reads. Between 86 and 98 percent of the reads mapped to the published reference sequence per sample (S5 Table). The final set of filtered SNPs consisted of 18,095 SNPs from 5,611 contigs (S6 Table). A high degree of heterozygosity was detected in both RRIM 600 and RRII 105 genotypes, 63% and 71%, respectively. While 22,421 SNPs from 8,395 contigs (from our assembly) were targeted, the differences between our assembly and the published rubber tree assembly made identification of the original SNP set too manually intensive to be feasible for such a large number of SNPs. This is because very few of the contigs to which probes were designed had a single blast match, likely reflecting the highly heterozygous nature of rubber tree clones which can cause problems with genome assembly software. However, every one of the 5,611 contigs with a SNP in the final set of filtered SNPs were listed as having a blast match (threshold 1e-06) to the contigs that the sequence capture probes were designed to, suggesting a high targeted SNP capture rate. So, despite the sequence capture probes being designed to a different reference, they resulted in a high number of useful SNPs that were then used to generate a linkage map.

### Linkage map

**Fig 1:**
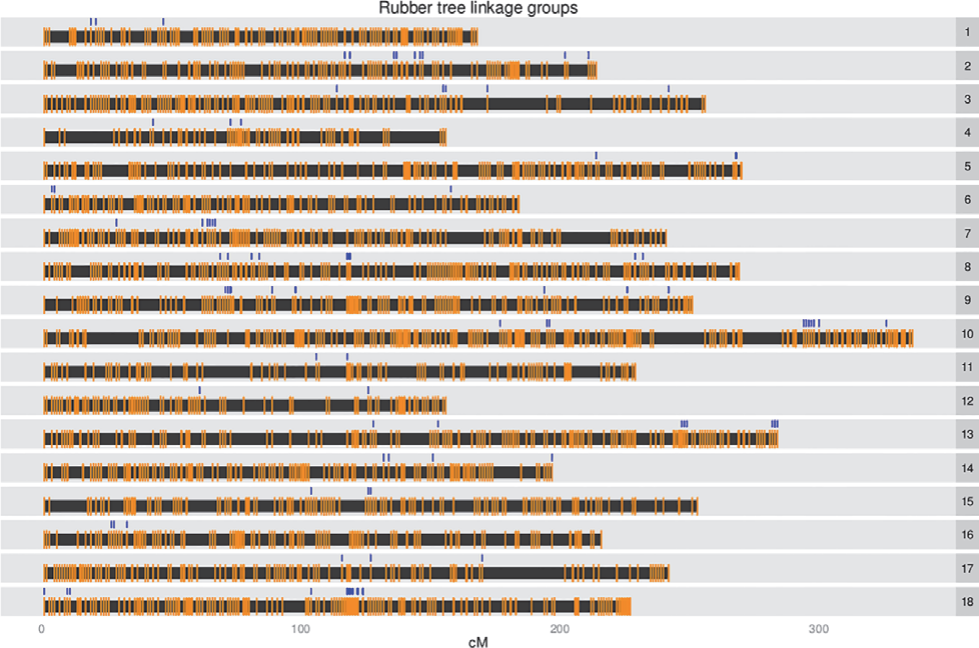
Rubber tree linkage group ideograms with positions of non-redundant markers indicated as orange vertical bars and markers from within placed scaffolds indicated as blue vertical bars

Linkage maps were generated using LOD thresholds from 8 to 18 with the map generated using a LOD threshold of 18 giving the most reliable result. Maps from lower LOD scores placed a large number of SNPs into a single linkage group resulting in less than 18 linkage groups. The LOD threshold of 18 gave a set of linkage groups most reflecting both the number of chromosomes found in the rubber tree and the linkage map generated from microsatellite data [20]. SNPs with redundant linkage information were not excluded as is typical in map construction because the aim was to place as many contigs as possible onto the map. There were 18 linkage groups and a total of 12,326 SNPs from 4,244 contigs could be placed on the linkage map (Table 1, Fig. 1). These contigs overlapped with 109 contigs that had been placed on the map by Rahman *et al*. [7] (S7 Table). Overlapping linkage groups were given the same linkage group number to be consistent with the literature. The smallest linkage group, LG4, was 156 cM and contained 319 SNPs or 60 non-redundant marker positions while the largest linkage group, LG10, was 336 cM and contained 1367 SNPs or 194 non-redundant marker positions (Table 1, Fig. 1). The median distance between markers (excluding redundant marker positions) was between 1.36 and 2.72 cM for each linkage group. The total map length was 4,160 cM, which appears high, but is similar to the estimated map length of PB 260, 3,428 cM, calculated using the Hulbert *et al*. method [20]. It is possible that genotype errors are causing the map to be longer than it should be, however, considering that the aim was to place contigs onto pseudo-chromosomes and that the results are consistent with previous linkage maps these errors do not affect the main result other than to highlight that a degree of uncertainty exists with the result, as it does with any similar study.

**Table 1:** Linkage groups, the number of SNPs they contain, the number of non-redundant marker positions and the total length in cM.

LG	SNPs	Non-redundant positions	Length (cM)
1	733	116	168.79
2	636	117	214.19
3	651	132	256.32
4	319	60	156.38
5	845	132	270.55
6	475	98	184.77
7	702	131	241.11
8	816	155	269.57
9	759	136	251.89
10	1367	194	336.85
11	435	79	229.08
12	448	88	156.72
13	780	142	284.83
14	834	124	197.99
15	609	121	253.95
16	519	104	216.93
17	496	106	242.47
18	902	151	227.95

There were 2,556 contigs with more than one SNP and 95% of these were within 4,500 bp of the nearest SNP with 22.6 kb as the greatest distance between SNPs. As such, SNPs within the same contig would be expected to place together on the linkage map and for the most part this is what was found with the median distance between SNPs within a single contig being 0 cM. However, there were 253 contigs where the distance between SNPs within that contig was greater than 10 cM and in several cases this distance was over 100 cM (S8 Table). Since these SNPs show normal segregation these contigs likely represent repeat sequences where the individual SNPs come from a single location. For example, the largest distance is 149.39 cM from contig AJJZ010937739.1 with nine SNPs, six SNPs at position 105.83 cM on linkage group 3 and 3 SNPs at position 255.22 cM, also on linkage group 3. The rubber tree genome is estimated at 2.15 Gb, yet the published reference genome accounts for 1.1 Gb, the remaining sequence would be composed partly of GC rich regions that PCR has trouble amplifying and largely of repeat sequence. Low copy number repeats can be collapsed into a single sequence during assembly while high copy number repeats can be collapsed or excluded completely depending on the assembly program. Thus, it is likely that many of the contigs from the published rubber tree genome will be low copy number repeat sequences.

Listing SNP location by contig allowed for scaffold structure to be interrogated, and for the most part confirmed. There were 707 scaffolds with SNPs in more than one of the constituent contigs and the median distance between contigs from within a single scaffold was 2.33 cM, consistent with the scaffold structure of the reference genome. There were 103 scaffolds with a gap between two of the constituent contigs at least 20 cM in size (S9 Table). Potential causes for this include recombination, repeat sequences causing assembly errors or genotyping errors resembling a recombination pattern. The most likely, given the repetitive nature of the rubber tree genome, is repeat sequences causing assembly errors. The sequence was originally mapped to contigs rather than scaffolds (in which joining contigs are separated by N's) to allow easier resolution of cases where contigs or scaffolds were placed into multiple linkage groups. Indeed, 16 cases were found where different contigs from within the same scaffold were uniquely assigned to different linkage groups (S10 Table). The cause for these discrepancies could be genotyping errors, or the contigs may be low copy number repeat sequences and occur in both locations with each SNP being unique to one of the locations (since they passed segregation analysis). One of these 16 scaffolds (scaffold 438547, which consists of 13 contigs AJJZ010977661.1 to AJJZ010977673.1) was placed in LG11 and LG4 each by a single SNP from two different contigs, AJJZ010977663.1 and AJJZ010977671.1, respectively. This scaffold also has microsatellite information from an additional contig that anchors the scaffold to LG11 (microsatellite mHbCIRa2535, Genbank: AY486752) which suggests correct placement is LG11 rather than LG4. The remaining 15 scaffolds only have SNP information, seven scaffolds have the majority of SNPs coming from a single linkage group making that the most likely placement while eight have a more equal SNP distribution between the conflicting placements (S10 Table).

There was one other contig with discordant placement, AJJZ010941895.1, that was placed in LG9 on our map by 3 SNPs, but in Rahman *et al*. [7] was placed in LG17 from scaffold 413909 which consists of 20 contigs (AJJZ010941882.1—AJJZ010941901.1) and was anchored using one microsatellite mHbCIRA2414 (Genbank: AY486700) that blasted to AJJZ010941893.1. Rahman *et al*. [7] placed the markers based on a blast sequence of the entire product, rather than just the sequence of the primers themselves (personal communication). The closest match for the forward primer is a partial match to contig AJJZ010048442.1 while the reverse primer has a perfect match to the end of contig AJJZ010672218.1. Alignment of the product sequence (Genbank: AY486700) shows a closer match between AJJZ010672218.1 than AJJZ010941893.1, yet this contig ends before the reverse primer (S1 Supporting Information) which may explain why AJJZ010941893.1 appeared as a better match in the result by Rahman *et al*. [7]. There were no SNPs in any of the other contigs from this scaffold, but the three segregating SNPs from contig AJJZ010941895.1 are within 2 cM of neighbouring SNPs, that, coupled with the blast match, is good evidence that this contig is placed correctly on our map.

The blast result of the primers that were used to make the linkage map by Le Guen *et al*. [20] identified three additional uniquely place loci that were not reported by Rahman *et al*. [7]. These were markers mHbCIRa169 (a.k.a A2410, Genbank: AY486877) [37], mHbCIRBAC12N03 (designed to Genbank: DQ115597) and mHbCIRMT67 (Genbank: AF383943) [20]. Marker mHbCIRBAC12N03 is from LG2 and both the forward and reverse primers blast to contig AJJZ011068040.1 (a.k.a scaffold 523829) (S11 Table). Marker mHbCIRa169 is from LG16, the forward primer blasts to contig AJJZ010333114.1 and the reverse primer blasts to contig AJJZ010333115.1, both of which are from scaffold 185402 which is composed of 6 contigs (AJJZ010333111.1—AJJZ010333116.1). Marker mHbCIRMT67 is from LG16, the forward primer blasts to contig AJJZ010860430.1 and the reverse primer blasts to contig AJJZ010860429.1, both of which are from scaffold 393529 which is composed of 23 contigs (AJJZ010860424.1—AJJZ010860446.1). Two of these blast results are confirmed by SNP placement in our linkage map: scaffold 185402 anchored to LG16 supported by two SNPs from contig AJJZ010333113.1; and scaffold 393529 anchored to LG8 supported by one SNP in each of contigs AJJZ010860436.1, AJJZ010860437.1 and AJJZ010860444.1. However, one SNP from contig AJJZ010860424.1 is assigned to LG2 suggesting that either this contig is misplaced in this scaffold, the SNP is misplaced in our linkage map or that contig AJJZ010860424.1 is a low copy number repeat sequence and occurs in both places.

While we were able to place 4,244 contigs from 2,909 scaffolds onto the map (S7 Table), including 3 contigs from the largest scaffold (scaffold 142103), many more than this can be placed by taking scaffold information into account. Adding the scaffold information allows for the placement of 23,712 contigs onto our linkage map. Including the above markers, mHbCIRA2414 and mHbCIRBAC12N03, and the scaffolds placed by Rahman *et al*. [7] that could not be placed using SNPs adds another 100 scaffolds containing 712 contigs. However, since these scaffolds cannot be anchored using SNPs their specific position can only be estimated relative to the scaffolds that have overlapping microsatellite and SNP placement. This gives a combined linkage map containing 24,424 contigs from 3,009 scaffolds spread over 18 linkage groups (S12 Table) and totalling 115 Mb (based on contig length), which is 10.4% of the total reference genome and includes 8,645 of the 20,639 intersect genes and 20,143 of the 69,300 union genes.

## Conclusion

We have used RNA-seq data from six rubber tree varieties to identify 368,594 variants from transcriptome sequence. We generated genome annotation files and used them to annotate the newly identified variants. We used target capture probes to enrich for a set of SNPs in a population of 149 rubber trees that were obtained through a cross between RRIM 600 and RRII 105 and used this information to generate a linkage map containing 12,326 SNPs from 4,244 contigs. Extending this map to incorporate scaffold information and map information from the literature results in a linkage map that includes 10% of the published reference genome and almost half of the functional gene content. This linkage map and the annotation files will be useful for gene mapping projects in the rubber tree and for improving the reference genome assembly.

## Supporting Information

S1 Dataset Contig sequences from BPM 24 to which the target capture probes were designed. (FASTA)

S2 Dataset Intersect gene set GFF. (7Z)

S3 Dataset Union gene set GFF. (7Z)

S4 Dataset cds of union gene set in fasta format. (7Z)

S5 Dataset Variants identified by RNA-Seq data (VCF file). (7Z)

S1 Supporting Information Alignment information for microsatellite mHbCIRA2414. (DOC)

S1 Table Annotation of Intersect gene set. (XLS)

S2 Table Annotation of Union gene set. (XLS)

S3 Table RNA-seq samples and read information. (XLS)

S4 Table RNA-seq variant summary type table from SNPeff result. (XLS)

S5 Table Ion torrent sample mapping metrics. (XLS)

S6 Table Final filtered SNP set and parental genotypes. (XLS)

S7 Table Linkage map markers indicating contig overlap with published map. (XLS)

S8 Table Contigs with discordant SNP placement. (XLS)

S9 Table Scaffolds with discordant contig placement. (XLS)

S10 Table Scaffolds with contigs that were placed into different linkage groups. (XLS)

S11 Table Microsatellite primer blast result. (XLS)

S12 Table Linkage map including scaffold and microsatellite information. (XLS)
