# Supplementary material for: SNP Identification from RNA Sequencing and Linkage Map Construction of Rubber Tree for Anchoring the Draft Genome
Source: PLoS One. 2015 Apr 1;10(4):e0121961. doi: 10.1371/journal.pone.0121961 (PMC4382108; doi:10.1371/journal.pone.0121961)
Supplement: S1 Supporting Information — (DOC) [file pone.0121961.s006.doc]

**S1 Supporting Information. Alignment information for microsatellite mHbCIRA2414**

Sequence alignment of the blast match between microsatellite mHbCIRA2414 (Genbank: AY486700) and contigs AJJZ010941893.1 and AJJZ010672218.1. The alignment of contig AJJZ010672218.1 finishes at the end of the GA repeat because this is the end of the contig sequence. The primer sequences are highlighted in yellow with mismatches indicated in red.

>AJJZ010941893.1 AACTGAAACCCACCTCACCAAACTAGTCCGAAAACTATAAATTCACAGATCAATCCCTCCAATCCTATCCCA

>gb|AY486700.1| AACTAAAACCCACCTCAAAAAACTAGTTCAAAAATCATAAATTCACGGATCAATCTCTCCACTCCTCTACCA

>AJJZ010672218.1 AACTAAAACCCACCTCAAAAAACTAGTTCAAAAATCATAAATTCACGGATCAATCTCTCCACTCCTCTACCA

>AJJZ010941893.1 AACAACCTCAGTTTATAGAGAGGAA-CTAGAGAGAGAAAAACCAAAACAAAAGCTTCCAACTACAGCAAAAGG

>gb|AY486700.1| -----CCTCAGTTTACAGAGAGGAAACTAGAGAGAGAAAAACCAAAACGAAAGCTTCCAACTACAGCAAAAGG

>AJJZ010672218.1 ---AACCTCAGTTTACAGAGAGGAAACTAGAGAGAGAAAAACCAAAACGAAAGCTTCCAACTACAGCAAAAGG

>AJJZ010941893.1 CAGAGAGAG-----------AGAGAGAGAGAAAGAGAGAGACAGAGG------GGTGTACTTTGGTCTGAGA

>gb|AY486700.1| CAGAGAGAGGTGGGGGGGGGAGAGAGAGAGAGAGAGAGAGAGAGAGAGAGAGAGGTGTATTCTGCCTTGGTG

>AJJZ010672218.1 CAGAGAGAG-----------AGAGAGAGAGAGAGAGAG(end of contig)

# Query: mHbCIRA2414_F

# Database: /Rubber_Tree/Genome/Contigs/Blast_db/AJJZ01

# Fields: query id, subject id, % identity, alignment length, mismatches, gap opens, q. start, q. end, s. start, s. end, evalue, bit score

# 195 hits found

mHbCIRA2414_F gi|445395777|gb|AJJZ010968025.1| 100.00 15 0 0 1 15 59 45 1.7 30.2

mHbCIRA2414_F gi|445408022|gb|AJJZ010959318.1| 100.00 15 0 0 1 15 1566 1580 1.7 30.2

mHbCIRA2414_F gi|447424978|gb|AJJZ010816392.1| 100.00 15 0 0 1 15 167 153 1.7 30.2

mHbCIRA2414_F gi|447535764|gb|AJJZ010705606.1| 100.00 15 0 0 1 15 546 560 1.7 30.2

mHbCIRA2414_F gi|447551748|gb|AJJZ010689622.1| 100.00 15 0 0 3 17 393 379 1.7 30.2

mHbCIRA2414_F gi|447577392|gb|AJJZ010664547.1| 100.00 15 0 0 1 15 256 242 1.7 30.2

mHbCIRA2414_F gi|447587027|gb|AJJZ010654912.1| 100.00 15 0 0 1 15 361 375 1.7 30.2

mHbCIRA2414_F gi|447596171|gb|AJJZ010645768.1| 100.00 15 0 0 1 15 191 205 1.7 30.2

mHbCIRA2414_F gi|447742585|gb|AJJZ010529599.1| 100.00 15 0 0 1 15 126 112 1.7 30.2

mHbCIRA2414_F gi|447804143|gb|AJJZ010468041.1| 100.00 15 0 0 1 15 300 314 1.7 30.2

# Query: mHbCIRA2414_R

# Database: /Rubber_Tree/Genome/Contigs/Blast_db/AJJZ01

# Fields: query id, subject id, % identity, alignment length, mismatches, gap opens, q. start, q. end, s. start, s. end, evalue, bit score

# 43 hits found

mHbCIRA2414_R gi|447569152|gb|AJJZ010672218.1| 100.00 18 0 0 1 18 6595 6612 0.028 36.2

mHbCIRA2414_R gi|445324276|gb|AJJZ011022073.1| 100.00 15 0 0 1 15 1082 1068 1.7 30.2

mHbCIRA2414_R gi|447610181|gb|AJJZ010632098.1| 100.00 15 0 0 1 15 271 257 1.7 30.2

mHbCIRA2414_R gi|447957364|gb|AJJZ010322282.1| 100.00 15 0 0 1 15 6047 6061 1.7 30.2

mHbCIRA2414_R gi|447993485|gb|AJJZ010286161.1| 100.00 15 0 0 1 15 238 252 1.7 30.2

mHbCIRA2414_R gi|447998365|gb|AJJZ010281281.1| 100.00 15 0 0 3 17 1349 1363 1.7 30.2

mHbCIRA2414_R gi|448047189|gb|AJJZ010232458.1| 100.00 15 0 0 1 15 7001 7015 1.7 30.2

mHbCIRA2414_R gi|445079813|gb|AJJZ011213616.1| 100.00 14 0 0 5 18 216 229 6.8 28.2

mHbCIRA2414_R gi|445194023|gb|AJJZ011125870.1| 100.00 14 0 0 2 15 70 57 6.8 28.2

mHbCIRA2414_R gi|445248782|gb|AJJZ011093192.1| 100.00 14 0 0 2 15 40 27 6.8 28.2
